# Temporally Selective Modification of the Tomato Rhizosphere and Root Microbiome by Volcanic Ash Fertilizer Containing Micronutrients

**DOI:** 10.1128/aem.00049-22

**Published:** 2022-03-21

**Authors:** Elijah C. Mehlferber, Kent F. McCue, Jon E. Ferrel, Britt Koskella, Rajnish Khanna

**Affiliations:** a University of California Berkeley, Department of Integrative Biology, Berkeley, California, USA; b USDA, Agricultural Research Service, Western Regional Research Center, Crop Improvement and Genetics Research Unit, Albany, California, USA; c Azomite Mineral Products, Inc., Nephi, Utah, USA; d i-Cultiver, Inc., Tracy, California, USA; e Department of Plant Biology, Carnegie Institution for Science, Stanford, California, USA; f The Chopra Foundation, Orlando, Florida, USA; University of Michigan-Ann Arbor

**Keywords:** micronutrients, root endophytes, volcanic ash, fertilizer, tomato microbiome

## Abstract

Food crops are grown with fertilizers containing nitrogen, phosphorus, and potassium (macronutrients) along with magnesium, calcium, boron, and zinc (micronutrients) at different ratios during their cultivation. Soil and plant-associated microbes have been implicated to promote plant growth, stress tolerance, and productivity. However, the high degree of variability across agricultural environments makes it difficult to assess the possible influences of nutrient fertilizers on these microbial communities. Uncovering the underlying mechanisms could lead us to achieve consistently improved food quality and productivity with minimal environmental impacts. For this purpose, we tested a commercially available fertilizer (surface-mined volcanic ash deposit Azomite) applied as a supplement to the normal fertilizer program of greenhouse-grown tomato plants. Because this treatment showed a significant increase in fruit production at measured intervals, we examined its impact on the composition of below-ground microbial communities, focusing on members identified as “core taxa” that were enriched in the rhizosphere and root endosphere compared to bulk soil and appeared above their predicted neutral distribution levels in control and treated samples. This analysis revealed that Azomite had little effect on microbial composition overall, but it had a significant, temporally selective influence on the core taxa. Changes in the composition of the core taxa were correlated with computationally inferred changes in functional pathway enrichment associated with carbohydrate metabolism, suggesting a shift in available microbial nutrients within the roots. This finding exemplifies how the nutrient environment can specifically alter the functional capacity of root-associated bacterial taxa, with the potential to improve crop productivity.

**IMPORTANCE** Various types of soil fertilizers are used routinely to increase crop yields globally. The effects of these treatments are assessed mainly by the benefits they provide in increased crop productivity. There exists a gap in our understanding of how soil fertilizers act on the plant-associated microbial communities. The underlying mechanisms of nutrient uptake are widely complex and, thus, difficult to evaluate fully but have critical influences on both soil and plant health. Here, we presented a systematic approach to analyzing the effects of fertilizer on core microbial communities in soil and plants, leading to predictable outcomes that can be empirically tested and used to develop simple and affordable field tests. The methods described here can be used for any fertilizer and crop system. Continued effort in advancing our understanding of how fertilizers affect plant and microbe relations is needed to advance scientific understanding and help growers make better-informed decisions.

## INTRODUCTION

The below-ground components of the plant support a diverse set of microbes that can interact intimately with their host. These bacteria are broadly split into two compartments, those surrounding the root surface (the rhizosphere) and those living inside the root (the endosphere). External inputs can cause changes in plant microbiome communities, which in turn may boost plant growth by controlling hormonal signaling, competing with pathogens, and increasing the bioavailability of nutrients (1–5). In particular, bacteria and fungi act to depolymerize and mineralize organic forms of N, P, and S, allowing for more efficient uptake by the plant (6–8). Plants also benefit from symbiotic relationships with mycorrhiza and nodulating bacteria, which help the plant to acquire nutrients more efficiently (9, 10). One of the primary factors that shape below-ground, plant-associated microbial community composition is the production of plant root exudates (11, 12). It is believed that these exudates are often targeted to attract beneficial bacteria which provide a fitness benefit for the plant (13). Such specifically recruited bacteria can provide benefits to the plant through disease suppression (14), increased nutrient acquisition (15), and/or improved resilience to abiotic stress (16). Furthermore, these bacteria can in turn modify the exudates that the plant is releasing, further altering the community and conditions of their local environment (17).

Understanding the factors that influence interactions between plants and below-ground microbial communities is critical to predicting the composition and function of the microbial community, especially in the context of agricultural practices of soil and plant amendments. These functions can be altered directly, through modifying the environment in which the bacteria are developing, as well as indirectly by modifying the interactions between the plant and its associated microbes. For example, studies have shown that microbial communities inhabiting agricultural soils can be impacted by nitrogen and phosphorus fertilizers (18, 19). Long-term use of N fertilizer reduced soil bacterial diversity, decreased soil pH, and reduced microbial P solubilization and mineralization capacity, whereas long-term use of P application increased microbial P immobilization (20, 21). N and P inputs have also been linked to specific changes in microbiome composition, with an increased abundance of copiotrophic microorganisms such as Proteobacteria compared to oligotrophic Acidobacteria (22–24).

The nutrient environment can also change a plant’s ability to control the composition and function of its associated communities. It has been shown in Arabidopsis thaliana that the phosphate stress response is linked to transcriptional regulators in the defense response, leading to a trade-off between phosphate starvation response and defense signaling, ultimately causing the development of an altered root-associated community (25). Specific inputs and practices introduced by the grower may thus lead to unintended consequences. In a controlled environment study, it was found that the addition of fertilizer abolished the ability of preinoculated tomato leaf-associated microbiota to protect against the pathogen Pseudomonas syringe (26).

Studies thus far have focused primarily on the role of major nutrients (N, P, K) in altering microbial community composition and dynamics, but relatively little work has been done to understand the effect of micronutrient amendments on the plant microbiome. Fortification of soil micronutrients is increasing in popularity because it can increase yield, fruit nutritional quality, and resilience to biotic and abiotic stress (27–30). In addition to B, Fe, Mn, Cu, Zn, and Mo, three other elements, selenium (Se), silicon (Si), and sodium (Na), have been shown to improve plant productivity and provide other benefits (31, 32). It is therefore important to understand how these rarer micronutrients affect the composition and function of plant-associated microbial communities.

In this work, we sought to better understand the role of micronutrient amendments in shaping below-ground microbial community composition. To do this, we used a popular commercially available amendment, Azomite, which is a surface-mined volcanic ash deposit, a Dacite by chemical composition. Azomite volcanic ash fertilizer has a silicon-based composition, which includes potassium, phosphorus, calcium, sodium, iron, magnesium, and manganese, along with trace amounts of zinc, copper, molybdenum, selenium, constituting over 50 rare earth elements identified by ICP (inductively coupled plasma) analysis. We have observed that this amendment increases fruit production, a result we quantified in this study. Given that this amendment impacts the productivity of the plant, we were interested in determining if, and to what extent, it might alter the associated microbial community. By looking at microbial community composition across bulk soil, rhizosphere, and root endosphere, we established that the amendment has a relatively small impact on community composition, but it has an impact on which bacteria seem to be the most closely associated with the plant. We present a novel approach to identify core microbes within a given microbial community being shaped by the combined effects of external and internal selective pressures. Using this new method, we identified groups of core taxa that shift in composition in response to Azomite treatment and suggest potential functional relevance for these changes.

## RESULTS

The purpose of this study was to determine whether a broadly used micronutrient soil supplement influences plant-associated microbial communities. We chose an amendment called Azomite (AZOMITE Mineral Products, Inc.), which has been used in plant and animal agriculture for decades. Azomite is commonly used by animal feed manufacturers to increase feed mill pellet production efficiency (33). It is also widely used in commercial crop production and is available at local garden stores as a general soil fertilizer. Treatment with Azomite was reported to reduce drought stress in greenhouse-grown tomatoes (34). Because of these considerations, we selected Azomite for this study to examine its impact on belowground microbiomes (soil, rhizosphere, and root endosphere) of greenhouse-grown tomatoes.

**FIG 1:**
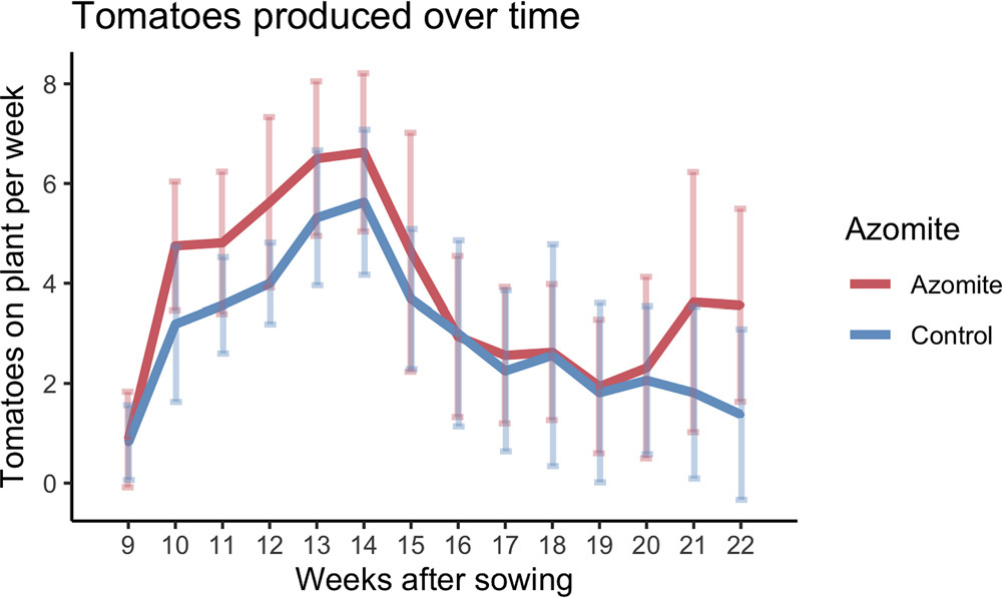
The effect of Azomite treatment on fruit production was determined by growing tomato plants (*n* = 32) in independent pots under controlled conditions for 22 weeks in a greenhouse setting. A standard fertilization regime was applied to both treated and control plants, with the treated plants being further supplemented with Azomite. The total number of tomatoes counted per plant weekly is plotted, showing a significant increase in the number of tomatoes on plants that were treated with Azomite, analyzed via ANOVA (*P* < 0.01). Tomatoes were harvested in weeks 15 and 18 for additional analyses, resulting in the reduction in the number of tomatoes counted in the following weeks.

To confirm our previous observations that Azomite increases fruit production (unpublished data), we grew an indeterminate (var. moneymaker) cultivar of greenhouse tomatoes in independent pots under controlled conditions for 22 weeks. As a supplement to the conventional nutrient program, we applied two different grades of Azomite in treated pots: granular, a coarser grade was mixed with soil, and ultrafine, a finer grade was applied later to the soil surface. Using a repeated-measures ANOVA with the number of tomatoes and weeks after sowing as main effects, and plant ID as a random effect, we found that treatment with Azomite significantly increased the total number of tomatoes produced per plant (*P* < 0.01) (Fig. 1). These data are consistent with reported increases in tomato yield on Azomite treated plants and are representative of other ongoing studies (E. Mehlferber et al., unpublished data). The observed increase in tomato production provided sufficient reason to examine potential changes in soil and plant microbiomes in response to Azomite application.

### Microbial diversity.

**FIG 2:**
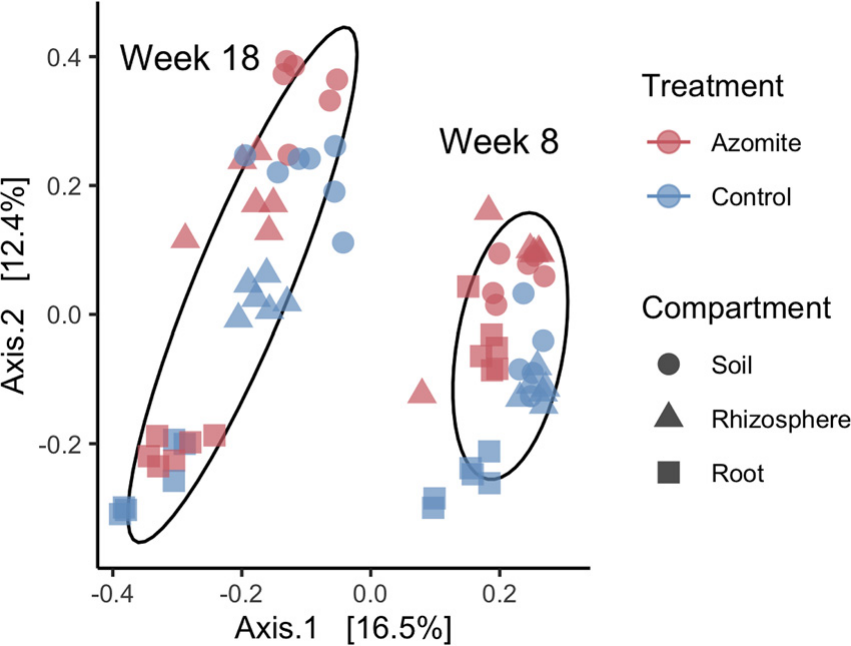
Principal coordinates analysis (PCoA) plot of Bray-Curtis dissimilarity among samples shows that community composition is driven primarily by compartment (bulk soil, rhizosphere, and root endosphere) explaining 14.6% of the variation, followed by time point (week 8 and week 18) explaining 12.2% of the variation, with a proportionally smaller effect of treatment (Azomite supplemented or control) explaining only 8.4% of the variation. Samples were relatively more distinct at later (week 18) sampling times than earlier (week 8) sampling times. An ADONIS test (nonparametric multivariate analysis of variance) indicated that each of these factors was significant (*P* = 0.001 for each), and that there were significant interactions between compartment and time point (*P* = 0.001), treatment, and time point (*P* = 0.001), as well as between compartment and treatment (*P* = 0.018) and that there was a significant three-way interaction between compartment, time point, and treatment (*P* = 0.033).

**FIG 3:**
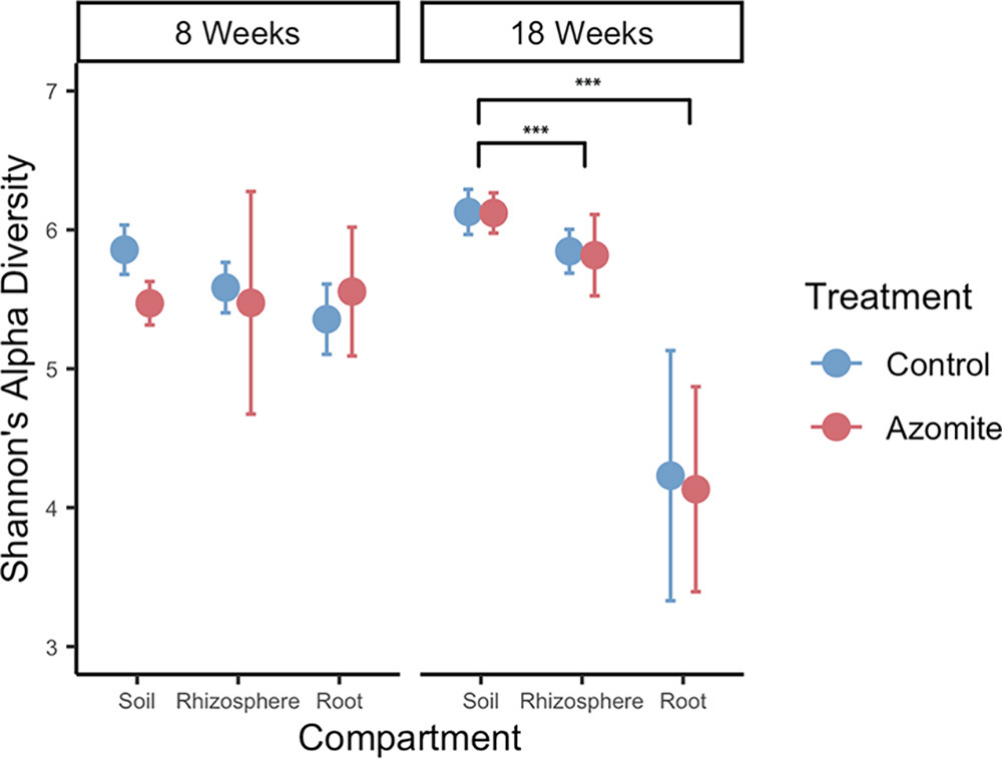
Shannon’s alpha diversity is shown for samples across a compartment (bulk soil, rhizosphere, and root endosphere), sampling time (week 8 and week 18), and treatment (Azomite supplemented or control). There is no significant effect of Azomite supplementation on the alpha diversity of the three compartments at either time point compared to the control plants. Both compartment (*P* < 0.001) and sampling time (*P* < 0. 001) were identified as significant factors in an ANOVA, but no significant difference was seen between the control and Azomite treated plants. A Tukey Post Hoc test was performed on the samples at each time point, indicating that the roots had significantly lower diversity at 8 weeks than the soil (*P* = 0.03) and that the roots were significantly less diverse than soil (*P* < 0.001) and rhizosphere (*P* < 0.001) samples at 18 weeks.

We examined microbial diversity in the three compartments (bulk soil, rhizosphere, and root endosphere) at two time points (week 8 and 18) in control and Azomite treated plants. We found that community beta-diversity was most impacted by the date, explaining 14.6% of the variation (*P* = 0.001), followed by compartment, explaining 12.2% of the variation (*P* = 0.001), and with finally treatment explaining only 8.4% of the variation (*P* = 0.001; Fig. 2). Furthermore, there were significant interactions between compartment and date (*P* = 0.001), and treatment and date (*P* = 0.001), as well as between compartment and treatment (*P* = 0.018) and a significant three-way interaction between compartment, date, and treatment (*P* = 0.033). This implies that bacterial communities changed in composition relatively independently over time in the soil, rhizosphere, and root endosphere, and while there was a significant effect of treatment, the amount of variation it explained was small. It should be noted that the amounts of Azomite applied to the bulk soil were relatively small (see Materials and Methods). Using an ANOVA test both compartment (*P* < 0.001) and sampling time (*P* < 0.001) were identified as significant, but there was no significant impact of Azomite treatment on alpha diversity. A Tukey post hoc test performed on data from each time point indicated that alpha diversity at 18 weeks was significantly lower in the roots than in both the soil (*P* < 0.001) and the rhizosphere (*P* < 0.001) (Fig. 3), suggesting that the root endosphere became more selective over time.

### Differential abundance.

Overall, as would be expected, time had a greater influence on differential abundance than treatment. Across all compartments, there were more differentially abundant taxa between weeks 8 and 18 samples than there were between Azomite and control (Fig. S1 and Table S1). Across time, the roots had the greatest number of differentially abundant taxa, followed by soil and rhizosphere (Fig. S1 and Table S1). Comparing between Azomite and control plants, the rhizosphere had the greatest number of differentially abundant taxa, followed by the roots, and finally the soil (Fig. S1 and Table S1).

### Abundance occupancy relationships.

We used the Sloan neutral community model (see Materials and Methods) to assess whether the relative abundance of taxa correlated with their relative percent occurrence across compartments. Based upon the neutral hypothesis, this model assumes that no other pressures are at work. Broadly, the abundance occupancy neutral model provided a good fit across the different samples, with a mean R^2^ value of 0.6899 for the soil, 0.7345 for the rhizosphere, and 0.7036 for the roots (Fig. S2). It is important to note that the model generally assumes free dispersal between samples, which may not be possible in this experiment due to each plant being separated into a pot in a greenhouse setting. The pots were proximally placed in a randomized order on benches in an enclosed greenhouse room. While we see some model changes fit over time, for example, the R^2^ value for Azomite treated root samples decreases from 0.7435 at 8 weeks to 0.5840 at 18 weeks, all values range between 0.5840 and 0.7689 and are generally consistent across time (Fig. S2). Information on individual taxa and their fit to the neutral model is available in Table S2.

### Azomite fertilizer associated taxa.

To examine possible taxa harbored by the Azomite fertilizer, we collected Azomite samples (as described) and analyzed the granular and ultrafine products that were used in the study. We identified 337 taxa in Azomite Granular (Table S3) and 248 taxa in Azomite Ultrafine (Table S4), there were a total of 435 unique taxa across both Azomite samples. We then compared these taxa to the bulk soil of control plants to determine which taxa were common to soil environments and which were likely introduced by the addition of the Azomite. Fig. S3 shows the mean relative abundance of these taxa across the compartments and treatments. Using paired t-tests between Azomite treated and control plants for each compartment, we determined that there was no significant difference in the relative abundance of Azomite originating taxa in any compartment. This indicated that the contributions that Azomite makes to shaping the microbiome were not due to the direct introduction of bacteria. Each plant was initiated with the same potting soil mixture (with or without Azomite), was exposed to the same fertilizer, pesticide program, and watering regime so that it could be assumed that the same bacteria were available to colonize each compartment in treated and control plants overall and this colonization would proceed randomly based on their initial abundance under a neutral model.

### Temporal evolution of core taxa.

Using the combination of differential abundance and abundance occupancy curves suggested in Shade and Stopnisek (35), we were able to identify a conservative set of “core” microbes in the rhizosphere and root endosphere and across treatment and time (Table S5). This scheme is illustrated in Fig. S4. First, we used DESeq2 (36) to identify microbes that were significantly enriched in the root endosphere and rhizosphere compared to the surrounding bulk soil (Fig. S4A, Table S6). These bacteria may be preferentially recruited by the plant from the bulk soil metacommunity, or these bacteria may preferentially occupy the compartment under the given conditions. These potentially enriched bacteria were then cross-referenced with the list of bacteria identified as being overrepresented in the occupancy abundance curve (Fig. S4B, Table S2), thereby identifying the bacteria as likely being important to the plant due to their relatively higher occupancy occurrence, than the bacteria expected based upon their relative abundance (Fig. S4C). It is important to note that these core taxa were not necessarily unique to the treatment in which they are identified as core, they may be found in other treatments, but do not meet the criteria to be identified as a core for that treatment.

**FIG 4:**
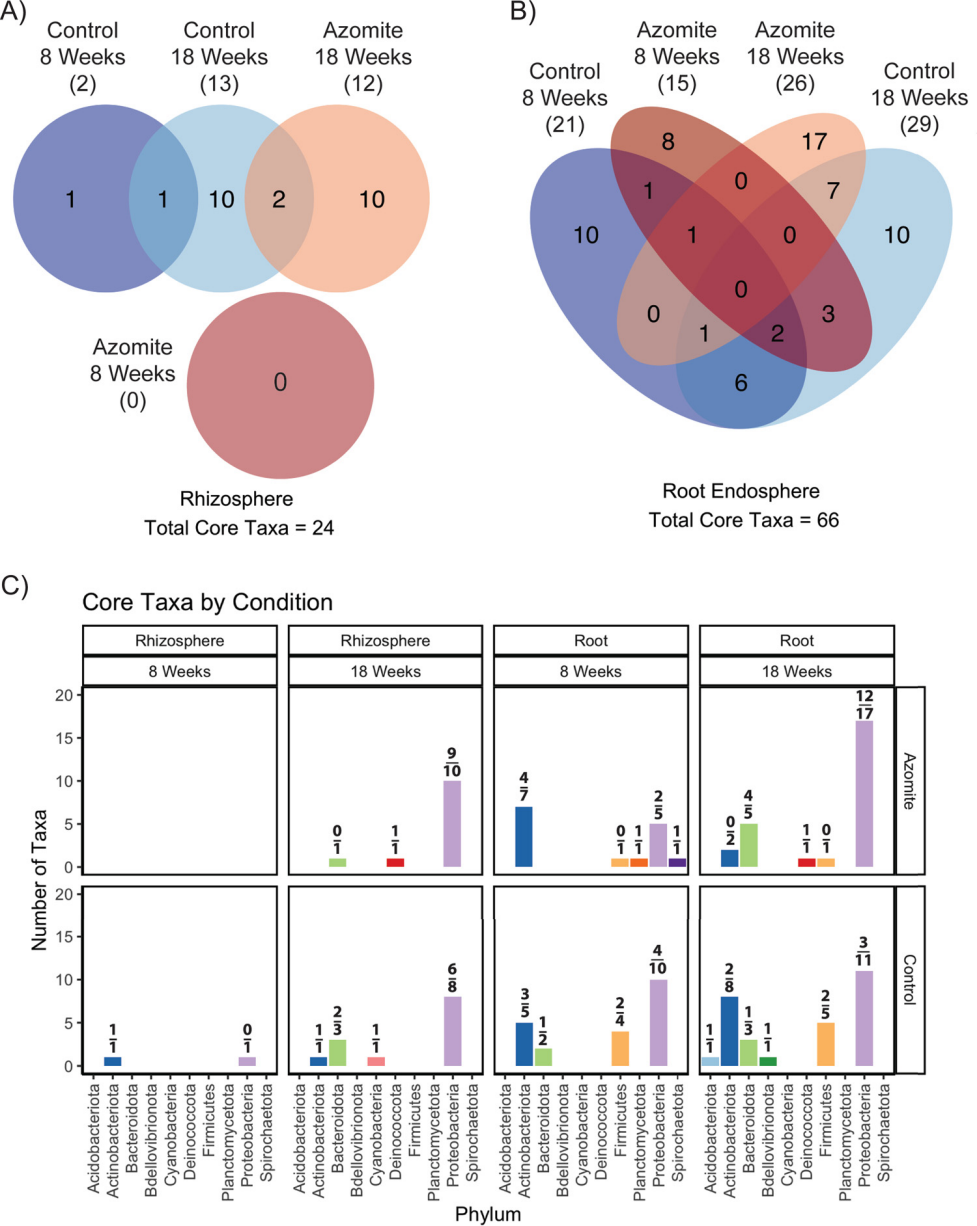
The “core” taxa under each condition were identified by determining which bacteria were both enriched in their associated compartment compared to the bulk soil and were at a significantly higher occupancy than would be predicted by their abundance using the Sloan neutral model. The Venn Diagram shows the overlap of the core portion of the microbiome across treatments and time for the rhizosphere (A) and root endosphere (B). Notably, no core taxa were shared across all three conditions in the rhizosphere (A). The number of core taxa identified is given in parentheses. The bar chart shows the number of taxa from each Phylum across treatments and time points (C). The ratio above each bar indicates the number of core taxa that were unique to only that treatment compared to the total core taxa for that phylum identified in the treatment.

We saw a stark difference between the rhizosphere and the root endosphere, with only 24 unique bacteria identified as “core” in the rhizosphere (across all treatments and time points) compared with 66 unique bacteria identified in the root endosphere as core taxa (Fig. 4). In the rhizosphere, these bacteria were primarily unique to their time point and treatment (Fig. 4A). Remarkably, 23 of the 24 rhizosphere core bacteria were identified in week 18 samples, with 10 unique to control and 10 unique to Azomite treated (Fig. 4A). Notably, only 1 unique taxon was identified in the control 8 weeks, and no taxa were identified as being core to the Azomite week 8 time point (Fig. 3A). These results suggested that the core rhizosphere community was being shaped between the tested time points of weeks 8 and 18 and that the core community did not coalesce until later in development (Fig. 4). Across both treatments, there was an increase in the number of Proteobacteria identified as the core from 8 weeks to 18 weeks (Fig. 4C).

There were appreciably more core taxa (66) identified in the root endosphere. Some of the core microbes were common between the sets, but the majority were unique to the time and treatment (Fig. 4B). Only 1/36 (2.8%) core taxon was common to all week 8 samples, and 7 (12.7%) core taxa were common to all week 18 samples, regardless of treatment (Fig. 4B). These data contrast the total taxa composition of the microbial communities, in which 45% of taxa were common to all treatments and time points in soil (168/370) and rhizosphere (170/370), and 39% (158/404) were common in all root samples (Fig. S5). We see that there is an increase in the number of Proteobacteria and Bacteroidota identified as core in the Azomite treated plants from 8 to 18 weeks, while the number of taxa from these groups remains relatively constant in the control. Likewise, we saw a decrease in the number of Actinobacteriota in the Azomite treated plants from 8 to 18 weeks, while we saw an increase in the control plants over that same period. We also saw a larger number of core Firmicutes in the control plants compared to the Azomite treated ones (Fig. 4C). Collectively, these data showed that the taxa identified as core by the method described above displayed relatively greater change over time than the total community composition, and particularly demonstrated that the Azomite week 18 root endosphere had the greatest shift in core taxa makeup.

### Predicted functions in the rhizosphere and root endosphere across treatments.

**FIG 5:**
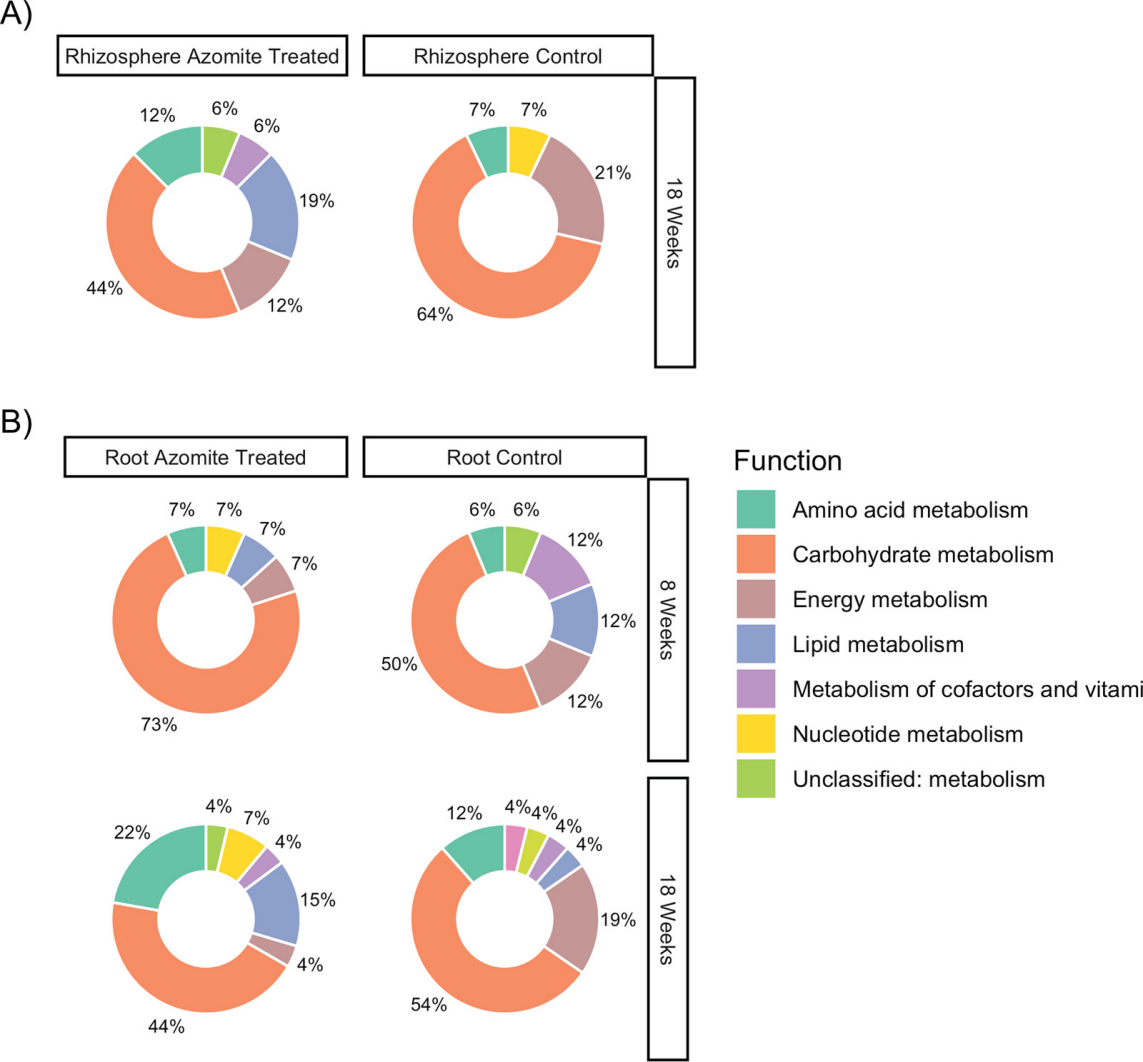
Potential functional differences were identified between the core microbiota across both time and condition by comparing gene content predictions from PICRUSt2. Pie Charts show the functional groups that were differentially abundant in each treatment and compartment as determined by DESeq2. The left panels show functions that increased in the Azomite treated plants, while the right panels indicate an increase in the control plants. Samples were separated by the compartment with week 18 in the rhizosphere (A), and weeks 8 and 18 in the root endosphere (B). Note that no bacteria were identified as being “core” in the rhizosphere at 8 weeks; therefore, no functions were shown.

We identified potential functional differences between the core microbiota across both time and condition by looking at the differentially abundant predicted genes from PICRUSt 2 (phylogenetic investigation of communities by reconstruction of unobserved states). Across all time points and treatments, the bulk of differentially abundant genes was related to carbohydrate metabolism (Fig. 5). This was followed by amino acid and lipid metabolism in the Azomite treated plants, particularly at 18 weeks, and by energy metabolism in the control plants (Fig. 5).

## DISCUSSION

Overall, these data indicate that the addition of the Azomite micronutrient supplement, shown to increase tomato yield (Fig. 1), played a subtle but important role in influencing the composition of the plant-associated microbial community. At the whole community level, we observed a small but significant impact on community composition measured through beta diversity with no significant change in alpha diversity (regardless of the compartment). This indicates that the community composition is shifting over time while retaining a similar level of richness, which is important given the noted contribution of species diversity in maintaining a healthy and disease-resistant community (37–40). The minimal impact of Azomite application on soil microbiota is not surprising since the amount of Azomite applied was relatively small (5% wt/wt of granular mixed in potting soil, and 1g per plant of ultrafine applied three times, every other week, to the base of the plant). These small amounts may not have been sufficient to cause any major shifts in the soil, but these treatments were enough to increase tomato production (Fig. 1) and influence the root endosphere composition over time with a greater change observed at the later time point (week 18). This also suggested that Azomite was not itself introducing new microbial taxa to the soil, as further exemplified by the fact that there were no significant differences in the relative abundance of taxa that may have been unique to the Azomite fertilizer across any treatment or compartment (Fig. S4).

Using DESeq2 we identified dozens of bacteria that are differentially abundant between the control and Azomite conditions across all treatments and time points. When comparing the Azomite and control plants we found a greater number of differentially abundant taxa in the rhizosphere and roots than in the bulk soil, indicating that the effect of micronutrients may be partially due to the altered interactions between the host plant and its associated microbiota. This could be due to shifts in plant available nutrients resulting in altered exudate profiles, a shift in immune signaling, and/or the result of the plant recruiting a different community to maximize its available environmental resources to support its developmental stage-specific growth and physiological processes.

We found minor differences in the R^2^ values associated with the abundance occupancy curves for each compartment and time point, with all values falling within the range (0.6 to 0.8), associated with a neutral assembly. Therefore, we can conclude that most bacterial communities found in each treatment and time point are likely to be shaped primarily by stochastic rather than deterministic forces. Despite this, some taxa in each treatment broke the assumptions of neutrality, providing further evidence that a portion of the community is being specifically recruited by the plant, either because of or aided by the current growing conditions.

Using a method first proposed by Shade and Stopnisek (35), we identified a core microbial community that was found to shift between time points and treatment, with most of the bacteria identified as core being unique (specifically being identified uniquely as core, they may still be present in other conditions) to their condition or time point. Given that this method was applied independently to replicate samples across different treatments, it provides further evidence that while most bacteria are not impacted by micronutrient addition, the bacteria that may be most important to the plant shift depending on the changes in its nutrient and metabolic needs over the developmental stages from early growth to maturity. This suggests that the plant-associated microbial community will shift as the plant adapts to changing conditions. The consequences of this shift warrant further study because these changes in community composition could have important implications for the plant’s survival and functioning.

Interestingly, fewer bacteria (24/370, 6%) were identified as being core in the rhizosphere compared to (66/404, 18%) in the root endosphere, with the Azomite week 8 condition having no taxa identified as the core. This could, in part, be attributed to the conservative nature of this test, which requires bacterial abundance and distribution to reach significance across two independent statistical tests. However, it also suggests that the plant is exerting greater control over the composition of its endosphere microbial community, a common observation as reviewed in Kandel et al. (41). This may also explain the compositional shifts we see in the core taxa, with an increase in Proteobacteria in the 18-week Azomite treated root samples. In the presence of the micronutrient, the plant may directly recruit these bacteria, or these bacteria may favorably occupy the altered compartment, or the micronutrient may cause changes in plant exudate compositions, which in turn may exert and influence these closely associated microbiotas, or, more likely, is some combination of the above. It is important to note that these changes in core taxa membership are likely a result of an interaction between Azomite application and plant developmental stage because the composition of the core community changes between time points even among the control plants.

**FIG 6:**
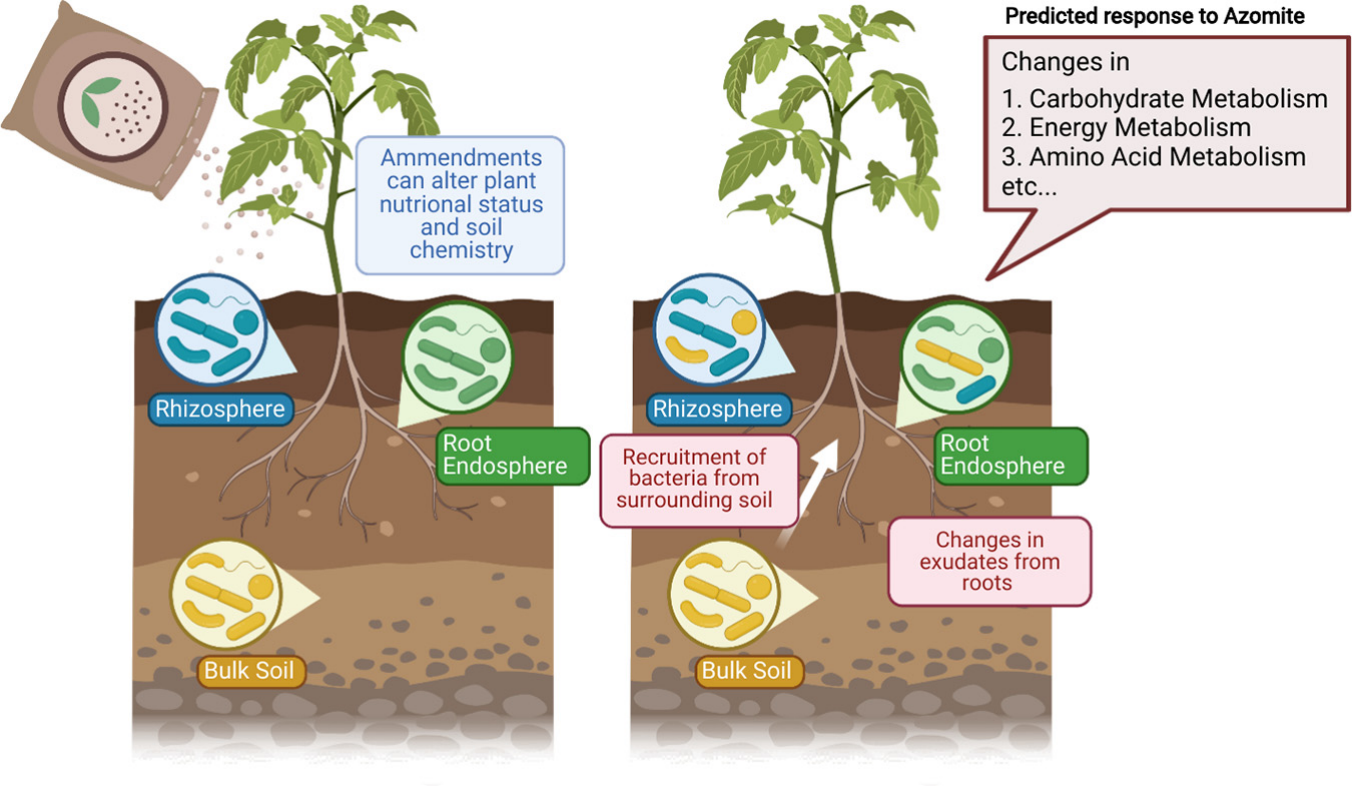
A working model showing the predicted impact of Azomite on the root microbiome. It was determined that the composition of the core taxa in the root endosphere changed differentially in response to the soil amendment. The change in composition showed a relative shift in most predicted core taxa functions, with most of these functions being related to carbohydrate metabolism, followed by energy metabolism and amino acid metabolism. These changes indicate that the treated roots may have altered exudate production, possibly due to changes in carbohydrate availability or type. This potential change in biochemical composition could act to recruit and attract different bacteria from the surrounding soil. The underlying mechanisms involved were being investigated. (Created with BioRender.com).

We present a working model (Fig. 6) to propose that induced changes in the plant’s production of endosomal substrates and possibly exudates in the presence of Azomite, cause the observed shift in root core taxa. We predicted that there would be functional differences between the Azomite and control communities, primarily in terms of carbohydrate metabolism (Fig. 5). This, along with the compositional differences between the core communities suggests that the substrates available to core taxa in the roots and late-stage rhizosphere may have changed in response to Azomite treatments (Fig. 6). Azomite contains nutrient elements, including potassium (K) and magnesium (Mg), which are essential for photosynthesis (42). Chlorophyll biosynthesis requires Mg, which is in the center of its porphyrin ring. Enrichment with accessible forms of K and Mg can promote photosynthesis, which could result in an increase in the availability of complex sugars to the root-associated bacteria. Indeed, preliminary (unpublished) evidence from a follow-up study suggested that Azomite utilization may be linked to plant photosynthetic capacity. Further studies are being performed to determine the mechanisms behind these predicted core functional changes and to determine if they represent a real shift in plant exudates.

The identified core bacteria warrant further investigation to conclusively determine the role that they play in the broader community. This method can be applied for preliminary identification of candidates, important bacterial taxa to be studied more closely for their roles in plant performance in different environments and response to soil inputs. Further studies on the functional relevance of the microbes recruited into the plant in response to Azomite alone and in combination with other treatments and other environments will provide us a better understanding of how plants, such as tomatoes, utilize available nutrients and other inputs to optimize their growth and development. It is anticipated that studies such as these will lead to the development of new approaches combining nutrient inputs with microbial amendments to improve agricultural outputs in both quantity and nutrient density of harvested specialty crops, like tomatoes. These combinatorial approaches are well poised to transform specialty crop production with potential economic benefits to growers and consumers.

## MATERIALS AND METHODS

### Plant generation and Azomite application.

Seeds of tomato (Solanum lycopersicum) variety moneymaker were surface sterilized by gently shaking in a solution of sodium hypochlorite and Tween 20 for 20 min, followed by two rinses with filter-sterilized H_2_O. Seeds were placed into soil pots and germinated in the greenhouse. When the seedlings were 3 to 4 in. tall, they were transplanted into larger pots, where they were grown for a total duration of 22 weeks with blocked placement on benches in a single greenhouse room. Plants were grown in the greenhouse under controlled conditions with supplemented lights to maintain long days and fans to control high-temperature fluctuations. Liquid nutrient supplementation program consisting of Peters Professional 20/20/20 water-soluble fertilizer was applied (1:64 ppm) once per week, as well as a disease suppression program consisting of Floramite and Decathlon at a rate of 1/4 tsp per gallon of water, mixed/agitated, was applied through a controlled sprayer at the rate of 1 to 2 gal per 100 plants. Sunshine mix number 1 (Sun Gro) containing 5% wt/wt chicken manure (EB Stone & Son, Inc.) was used either with or without 5% wt/wt Azomite Granular (AZOMITE Mineral Products, Inc.) for sowing and transplanting. After transplanting, the treated plants were supplemented with Azomite Ultrafine (AZOMITE Mineral Products, Inc.) by adding 1g per plant to the soil surface at the base of the plant at 7, 9, and 12 weeks after sowing. 16 plants each, either with or without Azomite fertilizer were grown and maintained through maturity. The number of red tomatoes produced per plant per week was quantified from week 9 until termination week 22.

### Sample collection.

Soil, rhizosphere, and root samples (as indicated) were collected from 6 control and 6 treated plants for microbiome profiling at two different time points: once at the onset of fruiting at week 8 and another one later during fruiting at week 18. Duplicate samples of Azomite Granular and Ultrafine fertilizer formulations were collected from their original packaging to determine whether any microbial taxa in treated soil could be linked to the Azomite product itself as the source. All samples were collected, as described in Deng et al. (27), on ice and kept frozen for DNA extraction.

### DNA extractions, 16s rRNA amplification, and sequencing.

DNA was extracted using the Qiagen DNeasy Powersoil kit. The V3-V4 region of the 16S ribosomal gene was amplified using a dual-indexed 16s rRNA Illumina iTags primer (341 F) (5′-CCTACGGGNBGCASCAG-3′) and 785 R (5′-GACTACNVGGGTATCTAATCC-3′) as described in Deng et al. (27). Samples were sequenced by the QB3 Vincent J. Coates Genomics Sequencing Laboratory facility at the University of California, Berkeley using Illumina Miseq. 300 bp pair-end with v3 chemistry.

### Data analysis.

Sequence files were demultiplexed and the adapters were removed by the sequencing facility. Raw reads were analyzed through the Dada2 pipeline in R using the default suggestions from their pipeline tutorial version 1.16 (43). Once amplicon sequence varients were produced in Dada2 we used Decontam to identify and remove contaminating sequences (43). Data were then imported into Phyloseq (44) for the remaining analysis. For differential abundance and occupancy abundance analysis, ASVs were merged at the genus level using the taxglom function in Phyloseq. We performed diversity analysis on unmerged ASVs but confirmed that the results are qualitatively unchanged when these tests are performed at the genus level. Using Bray-Curtis dissimilarity measures we performed an adonis test (nonparametric multivariate analysis of variance) on all samples together to determine the relative contribution of treatment (control versus Azomite-treated), compartment (soil, rhizosphere, root endosphere), and sampling time (8 or 18 weeks) on the composition of the microbiota. Alpha diversity was calculated using Shannon’s diversity index, which accounts for the richness (number of species) and evenness (distribution of species abundances within a sample) of each sample. We used an ANOVA to determine the impact of the various conditions on alpha diversity and then, after splitting by time point, a Tukey post hoc test to determine the differences between each compartment at each time point using adjusted *P*-values.

We used DESeq2 package to identify taxa at the genus level that show a significant difference in abundance across different conditions (Table S7). DESeq2 package was originally developed with the primary goal of identifying statistically significant variation in RNA seq data using a negative binomial distribution. However, to avoid the issues inherent in sample normalization McMurdie and Holmes (44) proposed its use in microbiome data analysis.

### Abundance occupancy curve.

To understand the forces that shape a microbial community it can be useful to assume a neutral hypothesis (that no forces are at work) and then evaluate how well this hypothesis matches the data. This can be done by utilizing the Sloan neutral community model developed by Sloan and Curtis (45, 46) and applied to plant-associated microbial communities by Shade and Stopnisek (35). This method fits the neutral model to the abundance-occupancy distribution under the assumption that bacteria that are more abundant (have a higher mean relative abundance) will have a correspondingly higher occupancy (percent occurrence across samples), given unlimited dispersal and equal fitness. The goodness of fit for this model can be used to determine the extent to which stochastic or deterministic forces are influencing the community composition, the former in cases where the model fits well, the latter when it fits poorly. The model can also be used to identify significant outliers, bacteria whose occupancy is likely to be influenced by some factors in the given environment. Bacteria that are significantly above the curve have a higher occupancy than expected given their abundance, and therefore are more likely to be selected for by the plant environment or may be exceptionally good dispersers. Meanwhile, bacteria that fall significantly below the curve are likely to have been selected against by the plant environment or have poor dispersal capabilities. Our model was constructed in R using rarified genus-level taxa tables and the function *fit_sncm* from the package *reltools* (47). Taxa were grouped at the genus level for two primary reasons, first, ASVs do not necessarily correspond to a single species because some species with multiple 16s RNA sequences will produce multiple ASVs, and second, we wanted to identify taxa that fill broadly similar ecological functions.

### Identifying core taxa in the rhizosphere and root endosphere.

We identified core taxa by the method proposed in Shade and Stopnisek (35), in which data from occupancy abundance curves and differential abundance were combined. First, we compared the relative bacterial abundance (at the genus level) between each compartment and the bulk soil using DESeq2 (with a threshold of *P* = 0.05) to determine which bacteria are at the significantly higher abundance and are potentially being selected for out of this meta-community reserve. Then, we compared this to the list of bacteria (at the genus level) that were identified as having significantly nonneutral (deterministic) occupancy, specifically those that appeared at higher-than-expected occupancy using *fit_sncm* (using taxa tables that were rarified to 40,000 reads per sample). The bacteria that were identified through both methods, which are at higher-than-expected occupancy and appear at the consistently higher relative abundance in the compartments associated with the plant, are more likely to be relevant to the plant under the given conditions as potentially beneficial microbes. It is important to note, however, that not all bacteria selected may represent beneficial species, but the bacteria selected by this process will include species that play mutually beneficial roles and are either host-selected or seek the present condition. This scheme was used to determine the Core Taxa independently for each sample (N = 6) under each condition, as illustrated in Fig. S1A to C. Core Taxa for each condition represent the bacteria that were present in abundance higher than randomly expected, and thereby form the core community in the host under that condition, implying that these species are likely to have been selected by the host, or they prefer the host environment under the tested condition. We repeated this analysis on ASVs that were not grouped at the genus level and saw comparable results in terms of core taxa membership (Fig. S6, Table S8).

### Functional analysis.

To better understand the compositional shifts in the predicted core taxa (merged at the genus level) across each condition we used PICRUSt2 (phylogenetic investigation of communities by reconstruction of unobserved states) set at default parameters to predict the gene family content of each core microbiome (48, 49). After the gene content was predicted by PICRUSt2, we used DESeq2 to determine which sets of predicted genes were differentially abundant between the Azomite treated and control core microbiota across the relevant time points and compartments. We used the PICRUSt2 function *add_descriptions.py* to link the genes to their Kegg Orthologs and these genes were cross-referenced against the KEGG (Kyoto Encyclopedia of Genes and Genomes) BRITE database (50–52) using the R function KEGGREST (53) to determine predicted functions. Functional analysis was also performed on ASVs that were not grouped at the genus level with comparable results (Fig. S7).

### Data availability.

Raw sequencing data have been submitted to the NCBI Sequence Read Archive (SRA) under the BioProject accession number PRJNA796360.
