# Supplementary material for: Temporally Selective Modification of the Tomato Rhizosphere and Root Microbiome by Volcanic Ash Fertilizer Containing Micronutrients
Source: Appl Environ Microbiol. 2022 Mar 21;88(7):e00049-22. doi: 10.1128/aem.00049-22 (PMC9004379; doi:10.1128/aem.00049-22)
Supplement: Supplemental file 1 — Fig. S1 to S7, Table S1, and descriptions of Tables S2 to S8. Download aem.00049-22-s0001.pdf, PDF file, 4.3 MB [file aem.00049-22-s0001.pdf]

Supplemental Figure 1

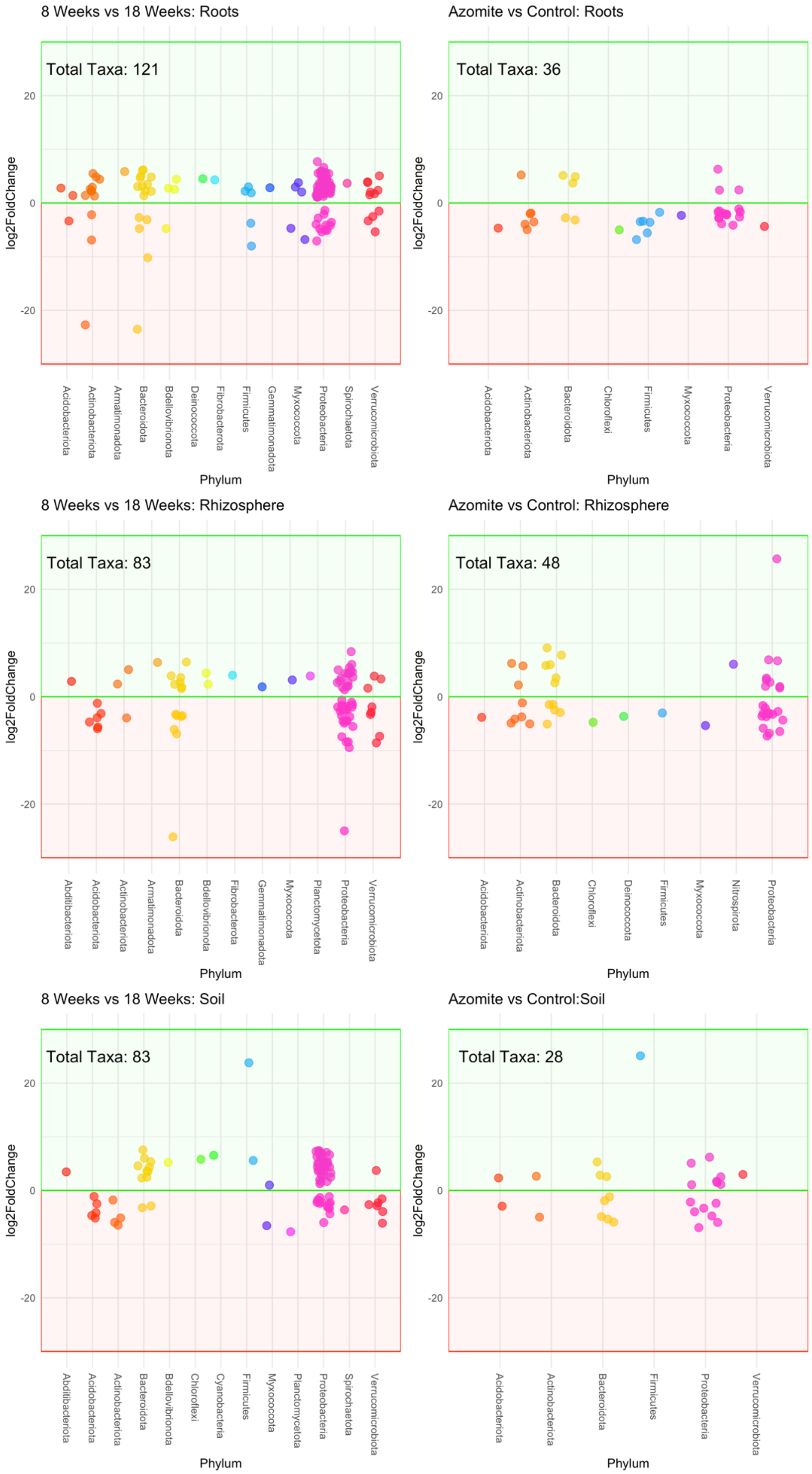

SM Fig 1

Significantly differentially abundant taxa in each compartment across time (left panels) and Azomite treatment (right panels), as identified by DESeq2. For the comparison between 8 and 18 weeks the taxa in the highlighted green portion of the graph are at increased abundance at 18 weeks compared to 8 weeks, and the taxa in red are at decreased abundance at 18 weeks when compared to 8 weeks. For the comparisons between Azomite and Control the taxa in green are at increased abundance in the Azomite treatment samples, while the taxa in the red box are at decreased abundance compared to the controls. Overall, there are more differentially abundant taxa when comparing across time than Azomite treatment, with the greatest number of taxa differing in the roots over time. See SM Table 1 for the numbers of taxa that increased or decreased in abundance.

Supplemental Figure 2

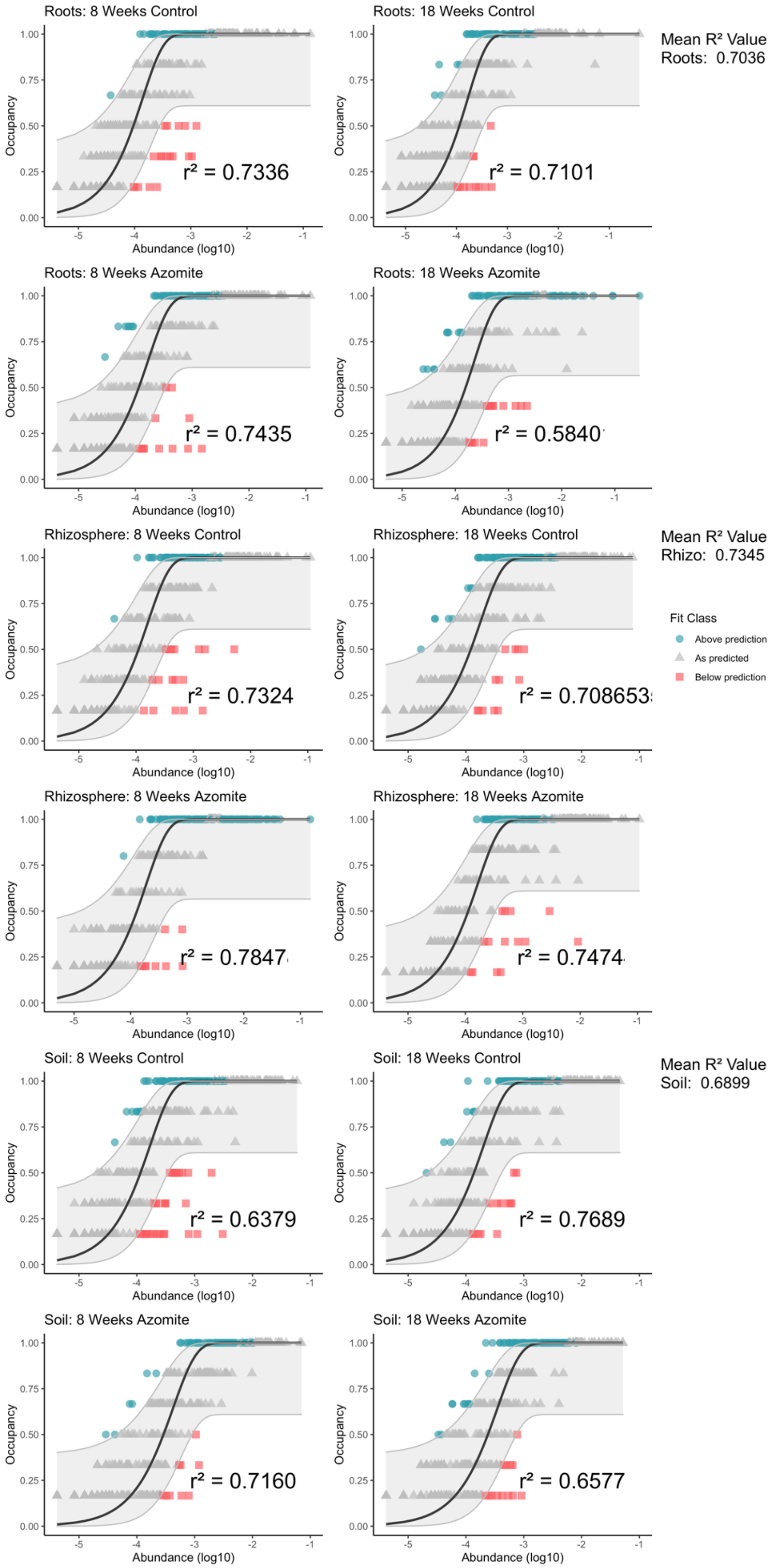

SM Fig 2
Abundance occupancy relationship and associated mean (r^2) values within each compartment across time and Azomite treatment. The line represents the predicted values based on the sloan neutral model, with a 95% confidence interval highlighted in grey. The taxa that fall above (blue) and below (red) their predicted values are highlighted and represent deviations from the neutral model and the possibility that they may be actively selected for (above the line, blue) or against (below the line, red). The mean R^2 values which represent the goodness of fit for the data to the predict slope of the neutral model are roughly similar across compartments.

Supplemental Figure 3

Relative abundance of taxa found in Azomite fertilizer but not bulk soil of control plants.

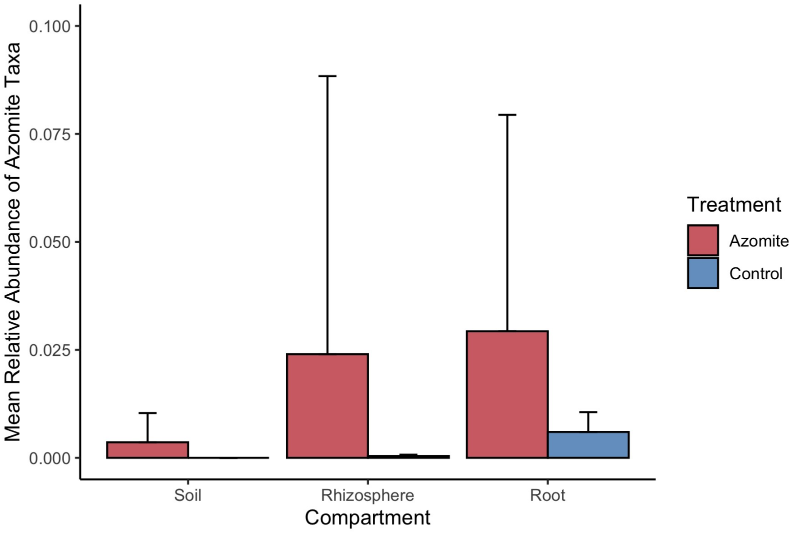

SM Fig 3
The mean relative abundance of taxa that were found to be present in the sequenced Azomite fertilizer, but not in the bulk soil of control plants. There was no significant difference in the relative abundance of these taxa across any of the compartments. In total, there were 435 unique taxa identified in the sequenced Azomite and 443 unique taxa in the bulk soil of control plants. Of those taxa, 272 were shared by both Azomite and bulk soil. Duplicate samples of Azomite Granular and Azomite Ultrafine collected from the original bags that were used in this study were subjected to 16s rRNA amplification and sequencing as described in Material and Methods. Genus level lists of the taxa present in Azomite Granular and Ultrafine are provided in SM Tables 2 and 3.

Supplemental Figure 4

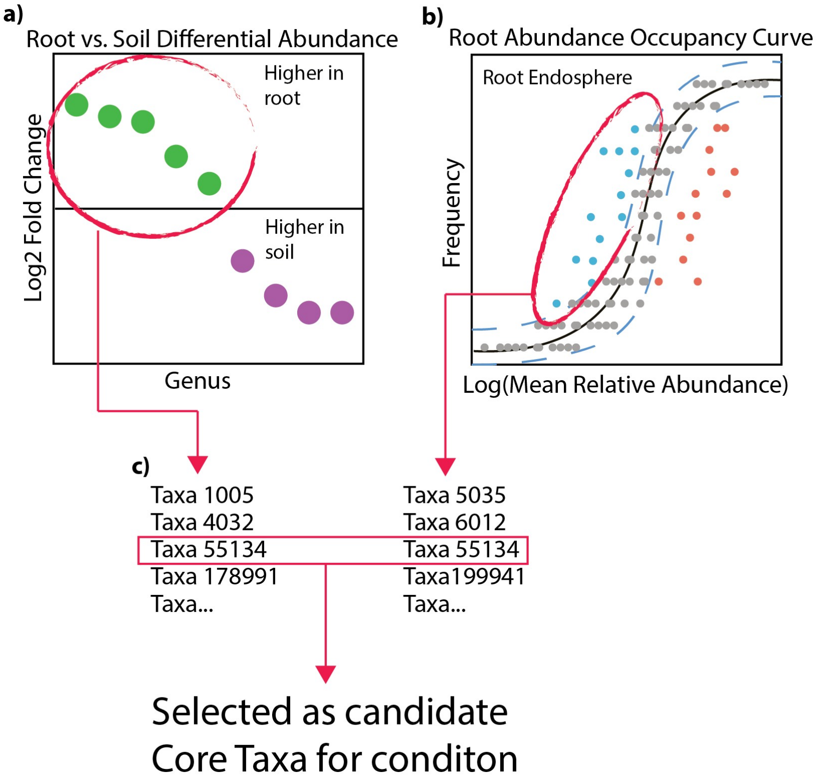

SM Fig 4
Methods used to identify the Core taxa in each condition are illustrated. Differential abundance is calculated between the root and soil samples of each condition using DESeq2 (a). Then, each taxon is plotted by its mean log10 relative abundance across these samples against its frequency, or the percentage of samples in this condition in which it is present. This is fit to the Sloan neutral model and those taxa that fall within the 95% confidence interval are considered to be neutrally selected (gray), while those that fall above or below are likely to be selected for (blue), or against (red), respectively by the plant or environment (b). The bacteria identified (a, green; and b, blue) are then cross referenced to select those that are significantly enriched and are above predicted frequency as the candidate “Core taxa” for the condition tested (c).

Supplemental Figure 5

Overlap between microbes in different compartments between control and treated samples at the two time points tested

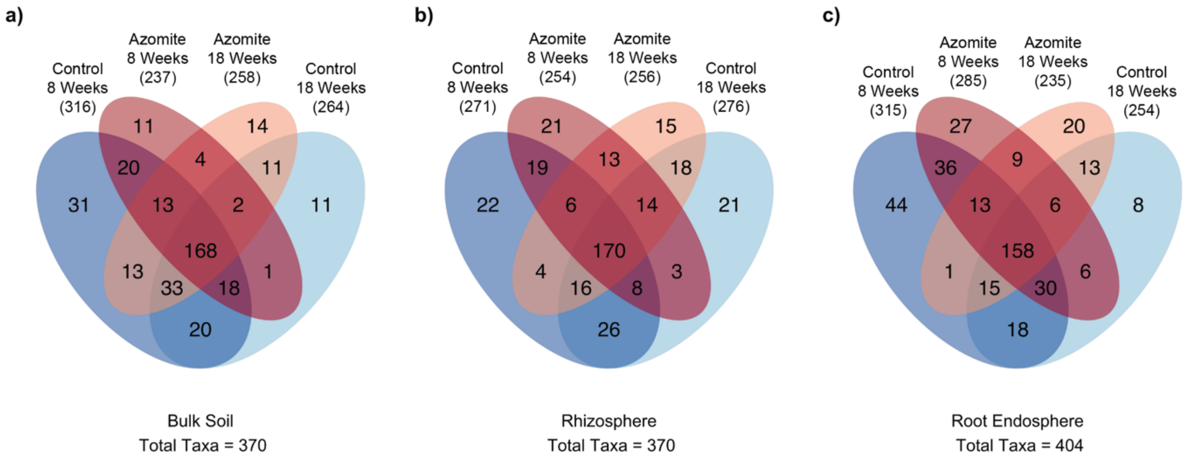

SM Fig 5
Total number of taxa shared in each compartment across treatment and time. Grouped at the genus level there were 370 total taxa across all bulk soil (a) and Rhizosphere treatments (b), and 404 in the Roots (c). Approximately half of the taxa in each compartment were present across all treatments and timepoints.

Supplemental Figure 6

Core taxa identified at the ASV level

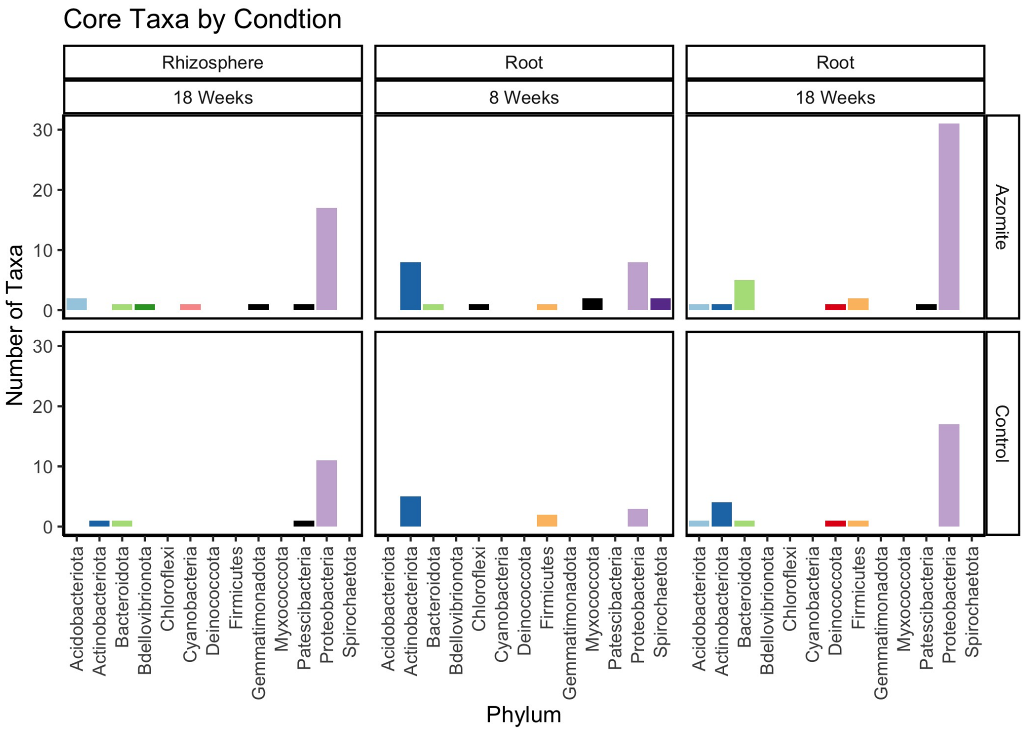

SM Fig 6
Number of core taxa in each Phylum across treatments and timepoints when the samples are not merged at the genus level prior to core microbe identification. Bars in black represent Phyla that are not found to be core in samples when analyzed at the genus level, but, overall, the patterns remain consistent regardless of sample processing.

Supplemental Figure 7

Functional differences identified at the ASV level

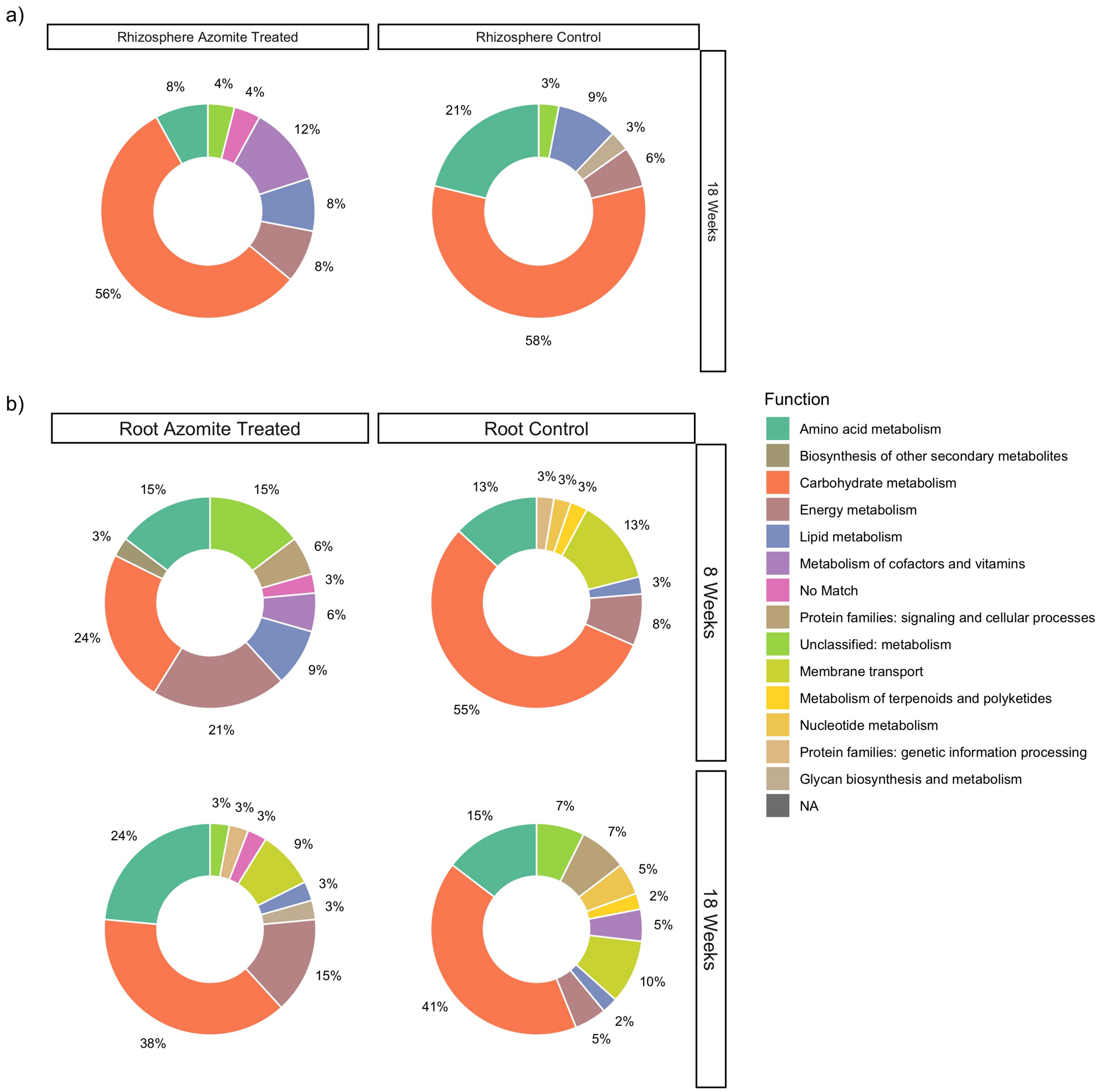

SM Fig 7
Pie Charts showing the functional groups that are differentially abundant in each treatment and compartment as determined by DESeq2 for samples that were not grouped at the genus level. Functions that were identified as more abundant in Azomite treated (left) and control (right) samples in the rhizosphere (a), and the root endosphere (b). As seen in the samples that are grouped at the genus level, the majority of differentially abundant functions are related to carbohydrate metabolism.

Supplemental Table 1.

Compartment	Comparison	Compared Across	Increased	Decreased	Total
**Roots**	Time	18 Weeks vs 8 Weeks	30	91	121
	Treatment	Azomite vs Control	7	29	36
**Rhizosphere**	Time	18 Weeks vs 8 Weeks	45	38	83
	Treatment	Azomite vs Control	19	29	48
**Soil**	Time	18 Weeks vs 8 Weeks	34	49	83
	Treatment	Azomite vs Control	14	14	28

Number of taxa exhibiting significant changes in their differential abundance in each compartment across time and in response to Azomite, as identified by DESeq2. Values of increase or decrease in abundance are plotted in SM Fig. 1
